# Determination of Photothermal and EMI Shielding Efficiency of Graphene–Silver Nanoparticle Composites Prepared under Low-Dose Gamma Irradiation

**DOI:** 10.3390/nano14110912

**Published:** 2024-05-23

**Authors:** Andjela Stefanović, Dejan Kepić, Miloš Momčilović, James L. Mead, Miroslav Huskić, Kamel Haddadi, Mohamed Sebbache, Biljana Todorović Marković, Svetlana Jovanović

**Affiliations:** 1 Vinča Institute of Nuclear Sciences, National Institute of the Republic of Serbia, University of Belgrade, P.O. Box 522, 11001 Belgrade, Serbia; 2 Faculty of Chemistry, University of Belgrade, Studentski trg 12-16, 11158 Belgrade, Serbia; 3 Department of Computing Science, University of Oldenburg, D-26129 Oldenburg, Germany; 4 Faculty of Polymer Technology, Ozare 19, 2380 Slovenj Gradec, Slovenia; 5 University of Lille, CNRS, University Polytechnique Hauts-de-France, UMR 8520-IEMN-Institut d’électronique de microélectronique et de nanotechnologie, F-59000 Lille, France; kamel.haddadi@univ-lille.fr (K.H.);

**Keywords:** silver nanoparticles, gamma irradiation, graphene oxide, electrochemically exfoliated graphene, photothermal efficiency, EMI shielding

## Abstract

Silver nanoparticles (Ag NPs) have been produced by low-dose (1–20 kGy) gamma irradiation of silver nitrate in the presence of graphene-based material (graphene oxide or electrochemically exfoliated graphene). The large surface area of those graphene-based materials combined with the presence of oxygen-containing functional groups on the surface provided successful nucleation and growth of Ag nanoparticles, which resulted in a uniformly covered graphene surface. The obtained Ag nanoparticles were spherical with a predominant size distribution of 10–50 nm for graphene oxide and 10–100 nm for electrochemically exfoliated graphene. The photothermal efficiency measurement showed a temperature increase upon exposure to a 532 nm laser for all samples and the highest photothermal efficiency was measured for the graphene oxide/Ag NP sample prepared at 5 kGy. Electromagnetic interference (EMI) shielding efficiency measurements showed poor shielding for the composites prepared with graphene oxide. On the other hand, all composites prepared with electrochemically exfoliated graphene showed EMI shielding to some extent, and the best performance was measured for the samples prepared at 5 and 20 kGy doses.

## 1. Introduction

Graphene, a one-atom-thick carbon sheet, has been attracting the attention of many researchers since its discovery in 2004 because of its unique structure. A perfect honeycomb crystal structure of graphene is composed of a carbon–carbon (C-C) sp^2^ hybridized network that forms a 2D planar sheet. In the carbon family, graphene has become a rising star due to its exceptional physicochemical characteristics, such as high surface area, low density, outstanding electrical conductivity, thermal stability, mechanical strength, and biocompatibility. In addition to its original features, graphene’s surface modification can provide additional functions and expand its range of applications. The material can be modified in terms of its physical and chemical characteristics, such as its preferred interaction with nearby species, improved mechanical strength, magnetic capabilities, catalytic qualities, and semiconducting behavior [1]. However, due to the disruption of its conjugated structure, the chemical modification of graphene affects its electrical conductivity [2]. To make graphene more water-dispersible, strong oxidants are often used to covalently modify graphene to produce graphene oxide (GO), which can later be reduced to obtain reduced graphene oxide (rGO) [3,4,5]. Strong oxidants covalently alter graphene’s structure to introduce various polar functional groups. The graphene-based lattice and existence of various oxygen-containing groups enable GO’s abundant fascinating properties. First, the functional groups on the GO surface serve as strong anchoring sites to immobilize a variety of active species. Typically, GO is insulating due to the large portion of sp^3^ hybridized carbon atoms domains and the presence of oxygen-containing groups. However, after the reduction of GO, the material turns into a semiconductor or even a semimetal similar to graphene [6]. Another way to produce graphene oxide is electrochemical exfoliation of graphite, which is a promising substitute since it decreases graphene’s degree of oxidation while maintaining its structural and electrical characteristics [7,8]. Briefly, the van der Waals forces in graphite are weakened by hydroxyl ions formed from the reduction of water during the electrochemical process, which bind to the edges of graphene and allow for the intercalation of electrolyte ions between graphene layers. In the following stage, intercalated ions are reduced, gas bubbles expand, and graphene layers separate. With this method, it is feasible to produce single- and few-layer graphene with a high yield and big flake size [9].

Due to its high surface area, graphene and its derivatives are great materials to anchor metal nanoparticles [10]. Those nanoparticles show unique optical, electronic, and chemical properties that are significantly distinctive from their bulk metal counterparts. The most extensively studied nanoparticles are noble metal nanoparticles that show promising results to be applied as sensors, fungicidal and bactericidal agents, in diagnostics and therapeutics, and in drug delivery, to list some [11,12,13]. An important characteristic of these nanoparticles is the existence of localized surface plasmon resonance (LSPR) phenomenon. Briefly, when the light of a specific wavelength interacts with electrons at the surface of the nanoparticle, their collective oscillation results in strong absorption of light as well as scattering. The kinetic energy of the oscillating electrons is then converted to heat through electron–phonon and phonon–phonon interactions and dissipated to the surrounding medium through particle–medium interfaces [14]. This phenomenon paved the way for the use of metal nanoparticles in water remediation, photothermal catalysis, and photothermal cancer therapy [15]. Another emerging application of these nanostructures is the development of electromagnetic interference (EMI) shielding materials owing to their high electric conductivity and large specific surface area. For this purpose, these nanostructures are often used as filling material with different polymers or carbon-based nanomaterials such as carbon nanotubes or graphene. For example, Kim et al. prepared a stretchable EMI shielding material of silver nanoparticles incorporated into microporous poly(styrene-b-butadiene-b-styrene) [16]. Zhang et al. incorporated Ag nanoparticles into a carbon nanotube sponge and reported the maximum EMI shielding efficiency of over 90 dB in the X-band with a 3 wt.% loading of Ag [17]. Li et al. covered a reflective layer of Al film with graphene/Ag nanoparticles coating and measured an EMI shielding effectiveness of 92.29 dB [18]. Nanowires of Ag were also investigated for EMI shielding applications, either sandwiched between graphene layers [19] or aligned and wrapped in graphene [20]. Apart from providing alternative pathways for electron transfer, graphene sheets act as barriers that block the contact of the Ag nanostructure with oxygen, which leads to a better stability of Ag nanostructures and a prolonged lifetime of the shielding material. 

Ag nanoparticles can be prepared by several methods: chemical reduction [21], spray pyrolysis [22], laser ablation [23], microwave plasma method [24], or UV light or electron irradiation [25,26]. Although the chemical reduction method is cheap and simple, it requires the presence of a stabilizing (capping) agent that prevents the overgrowth or agglomeration of nanoparticles and ensures their long-term stability. Ultrasonic spray pyrolysis provides control over particle size but demands high temperatures of up to 1000 °C. The physical methods for Ag nanoparticle synthesis have drawbacks such as high energy consumption and require high concentrations. Because it is straightforward, quick, and affordable, gamma irradiation offers an alternative to the traditional methods of creating silver nanoparticles [27]. It does not require high temperatures or extra reductants; thus, it is energy-efficient and environmentally friendly. Additionally, by performing the synthesis of Ag nanoparticles in the presence of graphene, the oxygen-containing groups on graphene’s sheets serve as locations where metal nanoparticles can be anchored [28]. Graphene then inhibits their oxidation and prevents nanoparticle agglomeration, thus making the use of stabilizing agents superfluous. To date, Ag nanoparticles were synthesized in the presence of graphene oxide or reduced graphene oxide. Hareesh et al. prepared a Ag-rGO nanocomposite with polyvinyl pyrrolidone employing gamma irradiation doses of 29, 58, 86, and 115 kGy [29]. A Ag-rGO nanocomposite with Roselle extract was prepared under 80 kGy and investigated for symmetric supercapacitor applications [30]. Liu et al. used the electron beam for the simultaneous reduction of GO and Ag^+^ ions employing doses from 70 to 500 kGy [31]. Kavitha et al. used lower doses of 2, 5, and 10 kGy to incorporate the Ag-GO composite into a glutaraldehyde (GA) crosslinked PVA matrix for radiation-sensitive optoelectronic applications [32]. However, the use of low doses of gamma irradiation for the preparation of Ag nanoparticles directly synthesized on graphene sheets and without additional stabilizing agents is insufficiently described in the literature. 

In this paper, we employed gamma irradiation at low doses (1–20 kGy) to obtain Ag nanoparticles anchored onto graphene sheets in a one-step synthetic procedure. Different microscopy and spectroscopy characterization techniques were used to examine the generated graphene/Ag NP composites, with a focus on the morphological and structural alterations brought on by gamma irradiation. The photothermal properties of the composites were determined by measuring the temperature changes under a 532 nm laser exposure. Furthermore, we measured the complex reflection and transmission of electromagnetic radiation up to 18 GHz and investigated their efficiency in EMI shielding.

## 2. Materials and Methods

### 2.1. Materials

Graphite powder (Sigma-Aldrich, St. Louis, MO, USA), highly oriented pyrolytic graphite rods (Vinča Institute of Nuclear Sciences, Belgrade, Serbia), concentrated sulfuric acid (Carlo Erba Reagents, Milano, Italy), sodium nitrate (Lach-Ner, Neratovice, Czech Republic), potassium permanganate (Merck, Darmstadt, Germany), hydrogen peroxide (Macron Fine Chemicals, Radnor, PA, USA), ammonium persulfate (Alfa Aesar, Ward Hill, MA, USA), and silver nitrate (Alfa Aesar) were used in this work. All reagents were used as received. 

### 2.2. Synthesis of GO/Ag NP and EEG/Ag NP Composites

As a starting graphene material, we used graphene oxide (GO) obtained by modified Hummers’ method and electrochemically exfoliated graphene (EEG) obtained by electrochemical exfoliation. To prepare GO, 2 g of graphite powder was mixed with 46 mL of concentrated H_2_SO_4_ and 1 g of NaNO_3_ and cooled to 0 °C. Then, 6 g of KMnO_4_ was added while the solution was continuously stirred in a water bath to keep the temperature below 20 °C. After 30 min of stirring, the temperature of the reaction mixture was raised to 35 °C and 100 mL of distilled water was added, after which the temperature was raised to 98 °C and maintained for 2 h. After that time, 400 mL of water was added to dilute the reaction mixture, and 2 mL of 30% H_2_O_2_ was added before allowing the liquid to cool to room temperature. The synthesized GO was purified by several cycles of centrifugation and washing with distilled water until the supernatant’s pH was neutral. Finally, the water was evaporated and removed, and GO was dried overnight at 60 °C in the oven. EEG was prepared in a two-electrode system using highly oriented pyrolytic graphite rods as both the counter and the working electrode, with a constant distance of 4 cm between the electrodes. Ammonium persulfate was dissolved in water to a concentration of 0.1 M to make the electrolyte solution. A direct current (DC) voltage of +12 V was applied, and the voltage was kept constant until the exfoliation process was finished, which was indicated by the total consumption of the working electrode. The exfoliated product was collected by vacuum filtration and thoroughly cleaned with deionized water to flush out any remaining salt. The product was then dispersed in water using an ultrasonic bath. The dispersion was centrifuged at 2575× *g* to remove any non-exfoliated graphitic material, and the supernatant was then utilized for the further steps. Graphene (GO or EEG) was dispersed in deionized MilliQ water using an ultrasonic bath to achieve stable dispersion with a graphene concentration of 1 mg/mL. Then, silver nitrate was added to a specific volume of the graphene dispersions to achieve an AgNO_3_ concentration of 0.001 M. As a scavenger of oxidative species produced during the radiolysis of water, isopropyl alcohol was added to the reaction mixture in a volume ratio of 1:10. Additionally, argon was purged through the reaction mixture before irradiation for 15 min to remove dissolved oxygen. The vials were then hermetically sealed and exposed to gamma radiation. Gamma-ray flux from the ^60^Co nuclide was used for the irradiations, with a dose rate of 8.8 kGy/h. Samples were exposed to the gamma irradiation source, receiving 1, 5, 10, and 20 kGy doses. After the irradiation, the samples were filtered (0.2 µm pore size, Isopore Membrane Filters, Darmstadt, Germany), rinsed with deionized water, and dried at 60 °C.

### 2.3. Characterization

Transmission electron microscope (TEM) examination of the samples was carried out using a JEOL (Tokyo, Japan) JEM-2100F using an acceleration voltage of 200 kV. The samples were dispersed in ethanol using an ultrasound bath and a drop of the mixture was placed on lacey carbon copper grids (200 mesh) and dried in the air. The particle size distributions were calculated using SemAfore software version 5.21. Scanning electron microscopy (SEM) analyses were performed on JEOL JSM-6390LV (Tokyo, Japan) microscope at room temperature. Powder samples were fixed on carbon adhesive tape. EDS measurements were performed on Oxford Instruments Aztec X-max (Abingdon, UK) energy-dispersive spectroscope. The LLG-uniSPEC 2 spectrophotometer was used to record the UV–Vis absorption spectra. To carry out the measurements, a small quantity of the dried material was dispersed in water, and spectra were recorded in quartz cuvettes at room temperature. Fourier-transform infrared spectroscopy (FTIR) was recorded on an Avatar 370 Thermo Nicolet spectrometer in the form of a KBr pellet. Thermogravimetric analysis (TGA) tests were performed on a TGA/DSC 3+ (Mettler Toledo instruments, Greifensee, Switzerland) under nitrogen (20 mL/min) at a heating rate of 5 °C/min, from 25 to 700 °C. Contact angle measurements were carried out by using the sessile drop method on the Theta Lite contact angle meter (Biolin Scientific, Gothenburg, Sweden). Thin films were made by passing 15 mL of composite water dispersion (concentration 1 mg/mL) through Merck Millipore 0.2 μm polycarbonate membrane using a vacuum. For data acquisition, ∼6 μL of deionized water (MilliQ 18.2 mΩ/cm) was carefully dropped on the samples using a micro syringe. All measurements were performed at ambient conditions (25 °C) and immediately after droplet stability. The data were analyzed using OneAttension software (version 4.0.3).

### 2.4. Photothermal Conversion Efficiency Determination

**Figure 1:**
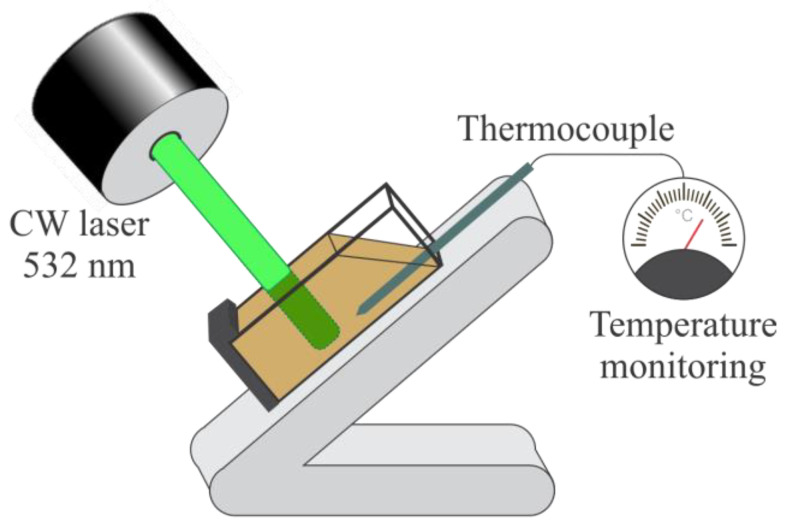
Experimental setup for the photothermal efficiency measurements.

Photothermal conversion efficiency measurements were performed in 1 × 1 × 4.5 cm spectrometer quartz cuvettes at room temperature (22.3 °C). Stable homogeneous dispersions of the samples in water with a concentration of 1 mg/mL were exposed to 532 nm continuous wave (CW) laser radiation. The laser power was 180 mW, the laser power density was 1.38 W/cm^2^, and the beam was circular with 1.5 mm in diameter. To ensure the perpendicular incident laser beam to the cuvette wall, a special cuvette holder was used (Figure 1). The center of the laser spot was placed at a fixed position in the center of the cuvette. The temperature evolution was recorded by a thermocouple (accuracy 0.1 °C) every 30 s. The samples were irradiated for 10 min (heating cycle) until they reached thermal equilibrium. Then, the laser was switched off to allow the sample to cool down to room temperature and the temperature was monitored for the following 15 min (cooling cycle). The photothermal efficiency of the samples was calculated using Roper’s method as previously described [33,34].

### 2.5. EMI Shielding Efficiency Measurements

Samples were prepared by passing 15 mL of 1 mg/mL water dispersions through 0.2 μm PC membrane using a vacuum. EMI shielding efficiency measurements were conducted using a Vector Network Analyzer (VNA) from Keysight Technologies (Streamline P5008A, Santa Rosa, CA, USA) operating in the frequency range 150 kHz–53 GHz. The VNA was connected through highly stable coaxial cables to a dedicated coaxial set-up to measure the complex reflection (S_11_) and transmission (S_21_) up to 18 GHz. The EMIs of the different samples are related to the residual RF signals transmitted through the shield. In other words, the amplitude of the transmission coefficient corresponds to RF signals that are neither reflected nor absorbed by the samples. Preliminary to the microwave characterization of the samples, vector calibration was performed at the output of the coaxial cables to remove systematic errors. Input RF power was set to −15 dBm, and intermediate frequency (IF) bandwidth was set to 100 Hz, resulting in a time per frequency of 10 ms. All measurements were conducted at room temperature. Each sample was sandwiched between two thin films of cellulose (named ‘’paper’’ in the following) to avoid any contamination of the coaxial flange.

## 3. Results and Discussion

The large specific area of graphene in the form of graphene oxide (GO) or electrochemically exfoliated graphene (EEG) was used as a support for the nucleation and growth of silver nanoparticles (Ag NPs) prepared by gamma irradiation at low doses (1–20 kGy). As a source of gamma irradiation, ^60^Co nuclide was used, which provides sufficient energy to cause the radiolysis of water and the emergence of reactive oxidative and reductive species. The primary reductive species that originate from the radiolysis of water such as hydrated electron (e^−^_(aq)_) and hydrogen radical (H^•^) have standard potentials of −2.9 V versus standard hydrogen electrode (SHE) and −2.4 V/SHE, respectively [35]. The introduction of isopropyl alcohol that acts as a scavenger of oxidative species helps in creating the predominately reductive environment, transforming the isopropanol molecule into a secondary radical α-methyl-hydroxyethyl radical [36]. Both primary and secondary species can reduce silver ions to a zero valent state considering that the standard redox potential for Ag^+^/Ag is 0.7996 V/SHE. 

**Figure 2:**
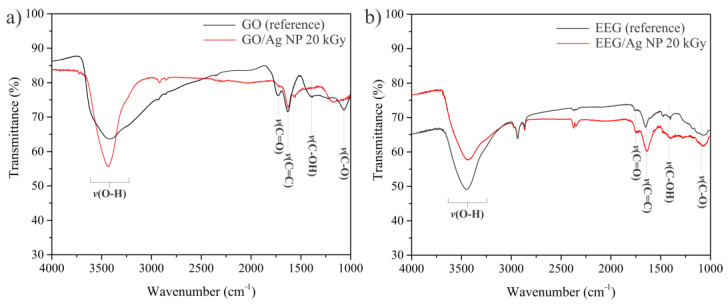
FTIR spectra of (**a**) GO (reference) and GO/Ag NP composite irradiated at 20 kGy and (**b**) EEG (reference) and EEG/Ag NP composite irradiated at 20 kGy.

The presence of oxygen moieties on graphene’s structure is responsible for the nucleation and growth of Ag nanoparticles [37,38,39], as well as for their stabilization after growth [40]. It is speculated that carboxyl and carbonyl groups are predominantly localized at the edges of sheets on sp^2^ hybridized C atoms, while hydroxyl and epoxy groups are placed on the basal plane on sp^3^ hybridized carbon [41]. In this work, we used two forms of graphene—GO obtained using strong oxidants by Hummers’ method and EEG prepared under mild conditions from highly oriented pyrolytic graphite. To inspect the presence and type of oxygen functional groups attached to graphene sheets before and after the irradiation, FTIR analysis was performed. As can be seen from Figure 2, both types of graphene show similar absorption bands that can be attributed to various oxygen-containing functional moieties on the graphene’s surface. The broad band at 3420–3450 cm^−1^ originates from the O-H stretching vibrations of the C-OH groups and adsorbed water molecules, while the bands at 1717, 1635, 1385, and 1060 cm^−1^ correspond to the stretching vibrations from carbonyl groups, stretching vibration of C=C bond, stretching vibrations of hydroxyl or carboxyl groups, and stretching vibrations of C-O groups, respectively. After the irradiation, in the spectrum of GO/Ag NPs, bands at 1385 and 1060 cm^−1^ are not detectable, while the band at 1717 cm^−1^ from carbonyl groups has decreased intensity. On the other hand, the spectrum of EEG/Ag NPs shows the same bands as the starting EEG. This might be an indication of a better susceptibility to the reduction of GO under gamma-ray flux in comparison to EEG. 

**Figure 3:**
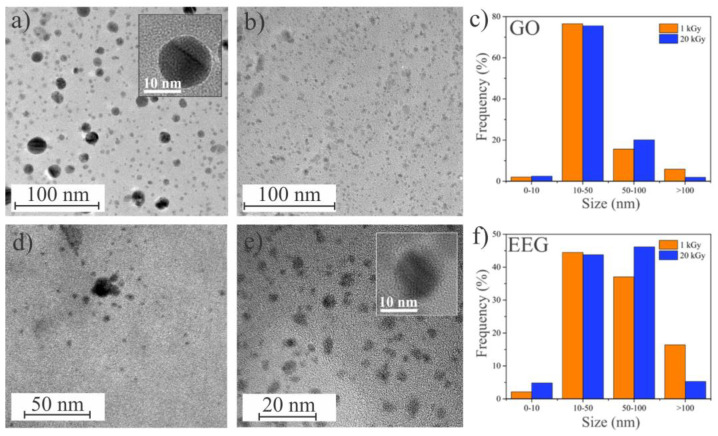
TEM images of (**a**) GO/Ag NPs prepared at 1 kGy irradiation dose, (**b**) GO/Ag NPs prepared at 20 kGy irradiation dose, (**c**) particle size distribution for GO/Ag NPs, (**d**) TEM images of EEG/Ag NPs prepared at 1 kGy irradiation dose, (**e**) EEG/Ag NPs prepared at 20 kGy irradiation dose and (**f**) particle size distribution for EEG/Ag NPs.

The nucleation and growth of Ag NPs depend on the number of oxygen-containing functional groups that have the role of nucleation sites. The high density of these groups favors nucleation over growth, which consequently leads to a higher number of Ag nanoparticles [37]. In the opposite case, the low density of these groups is advantageous to growth, which yields a smaller number of these nanoparticles but comparably bigger. To provide a better insight into Ag nanoparticle size, we recorded TEM images of the GO/Ag NPs and EEG/Ag NPs samples irradiated by the lowest and the highest applied dose (Figure 3). TEM provides Z-contrast images which enable a clear distinction between Ag (Z = 47) that appears as dark spots compared to pale gray areas of C (Z = 6). For both GO and EEG, the obtained Ag nanoparticles are predominantly spherical and uniformly cover the surface of graphene sheets. Both types of graphene successfully prevented the agglomeration and creation of large aggregates of Ag nanoparticles that commonly occur when Ag nanoparticles are synthesized without additional stabilizers. For GO, the majority of obtained Ag nanoparticles (~75%) have sizes between 10 and 50 nm, while for the applied dose of 20 kGy, we noticed only a minor increase in the 50–100 nm particle size range and a decrease in big particles (>100 nm). On the other hand, only 45% of Ag nanoparticles prepared on EEG have sizes between 10 and 50 nm and a significant portion of Ag nanoparticles is larger. This dissimilarity in Ag nanoparticle size distribution might be due to the difference in the number of oxygen-containing functional groups between GO and EEG. Additionally, for EEG, a considerable increase in 50–100 nm particles and a decrease in >100 nm particles could be noticed after the 20 kGy dose irradiation.

**Figure 4:**
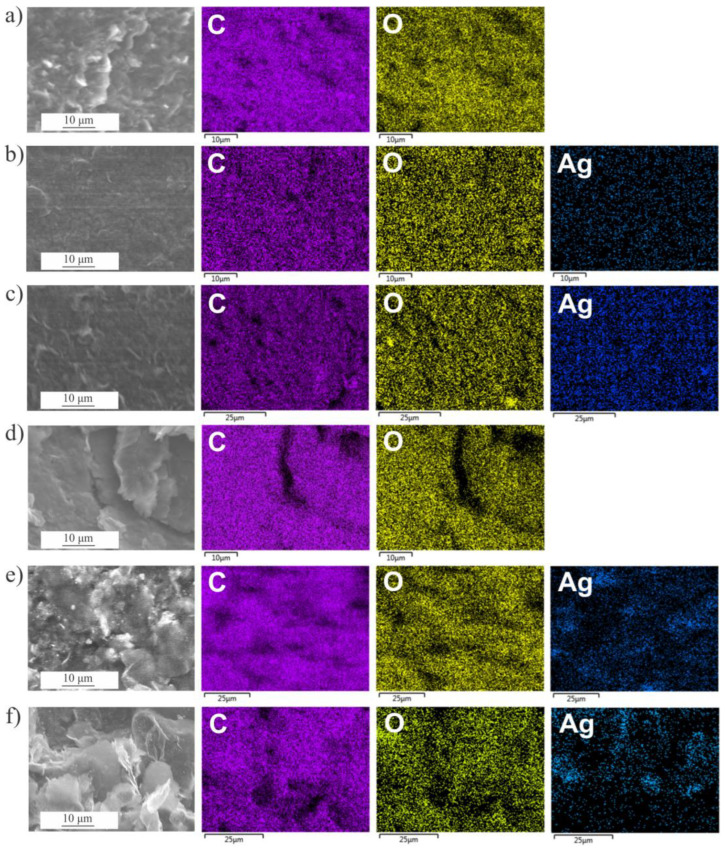
SEM image and EDS maps of the corresponding area for carbon, oxygen, and silver for (**a**) GO, (**b**) GO/Ag NPs prepared at 5 kGy, (**c**) GO/Ag NPs prepared at 20 kGy, (**d**) EEG, (**e**) EEG/Ag NPs prepared at 5 kGy, and (**f**) EEG/Ag NPs prepared at 20 kGy.

SEM-EDS analyses were performed to explore the distribution of the constituent elements of the prepared composites (Figure 4). In the starting graphene materials (GO and EEG), we detected only C and O with traces amount of sulfur and sodium that remained from the synthetic procedure. Elemental analysis showed that GO has a higher O content than EEG (Table 1). All composites of GO and EEG showed homogeneous coverage of graphene sheets by Ag nanoparticles. 

**Figure 5:**
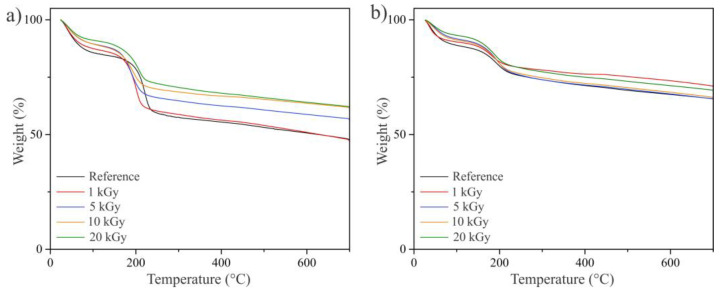
TGA curves of (**a**) GO (reference) and GO/Ag NPs prepared at different irradiation doses and (**b**) EEG (reference) and EEG/Ag NPs prepared at different irradiation doses.

The difference in oxygen-containing functional group abundance between GO and EEG is well depicted in the TGA curves (Figure 5). The samples were firstly heated to 700 °C in a nitrogen atmosphere. The TGA graphs showed three temperature zones with distinctive mass loss steps: region (100 °C) associated with the loss of adsorbed moisture, region (100–360 °C) associated with the breakdown of thermally labile oxygen-containing groups, and region (360–700 °C) associated with the decomposition of the carbon lattice [42,43]. EEG shows greater thermal stability and maintains 65.8% of the weight at 700 °C compared to GO (48.5% of the weight at 700 °C). This is a consequence of a greater portion of thermally labile oxygen-containing groups in GO than in EEG. In addition, for both GO and EEG, weight loss is most prominent for non-irradiated samples, and with the increase in the irradiation dose, this weight loss gradually decreases. This is an indication of the partial restoration of graphene’s conjugated structure and the elimination of oxygen-containing functionalities caused by the reduction under gamma irradiation [27].

**Figure 6:**
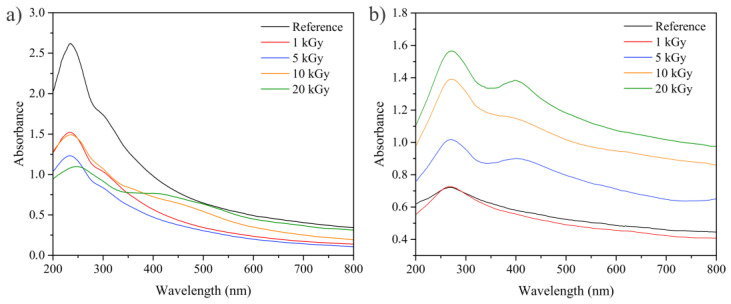
UV–Vis spectra of (**a**) GO (reference) and GO/Ag NPs prepared at different irradiation doses and (**b**) EEG (reference) and EEG/Ag NPs prepared at different irradiation doses.

The partial restoration of graphene’s conjugated structure can be followed by UV–Vis spectroscopy (Figure 6). The spectrum of GO displays two different features: a strong peak at 230 nm resulting from the aromatic C=C bonds’ π-π* transition, and a shoulder at ~300 nm resulting from the C=O bonds’ n-π* transition, while the spectrum of EEG shows only π-π* transition of aromatic C=C bonds at 270 nm. The peak from the aromatic C=C bonds shows a gradual redshift with the increase in the irradiation dose, while the shoulder at ~300 nm in the GO/Ag NPs samples gradually decreases until it completely vanishes at the higher applied doses of 10 and 20 kGy. In addition, Ag NPs are characterized by the presence of a localized surface plasmon resonance (LSPR) peak as a consequence of the collective oscillation of electrons from the conduction band with respect to the lattice of positive nuclei. This LSPR peak appears as one broad peak at ~420 nm in GO/Ag NPs samples and ~400 nm in EEG/Ag NPs samples, and it is most distinguishable for the highest applied dose. The difference in the LSPR peak position might be induced by the variation in Ag nanoparticle sizes prepared with GO and EEG.

**Figure 7:**
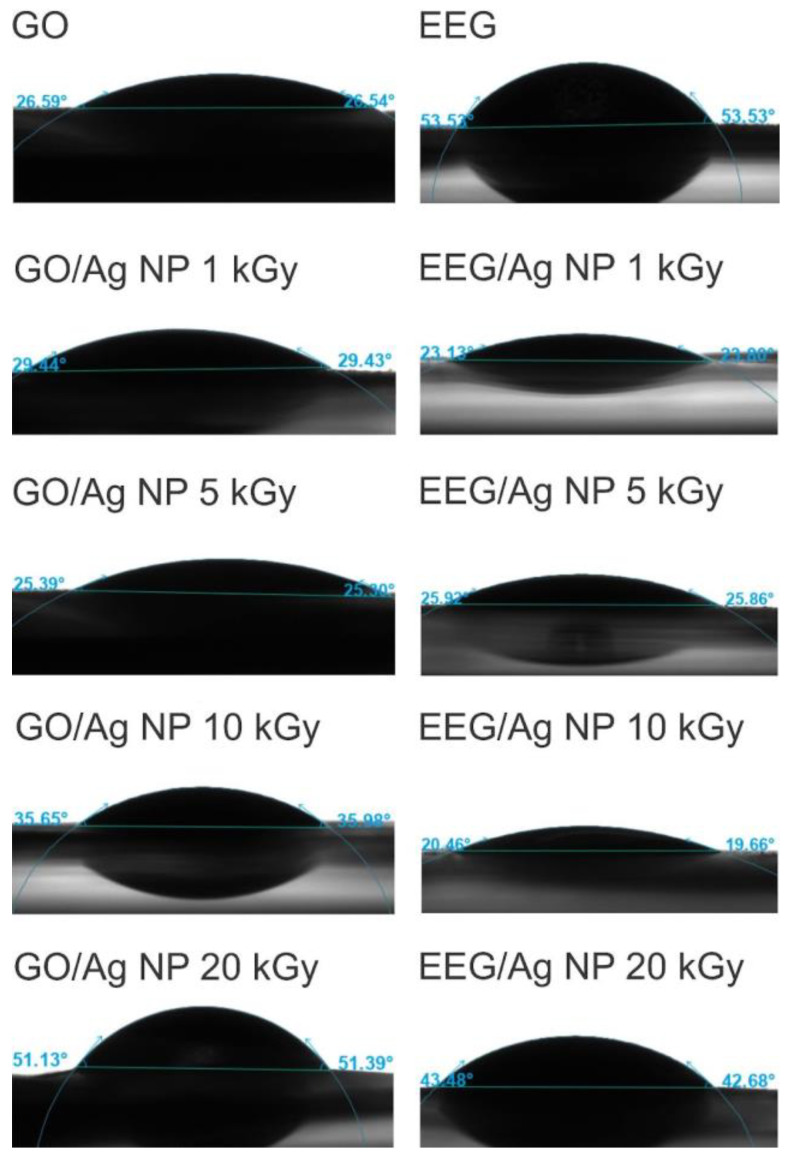
The contact angle of the studied graphene/Ag NPs thin films on polycarbonate membrane.

The wettability of composite thin films prepared by vacuum filtration was investigated by measuring the contact angle of water droplets (Figure 7). As expected, both pristine GO and EEG show hydrophilic behavior with contact angles of 26.5° and 53.5°, respectively. The higher measured contact angle for pristine EEG compared to GO is a result of the lower abundance of polar oxygen-containing groups on its surface. GO/Ag NP composites show a slight increase in contact angle until reaching the maximum of 51.2° for the composite irradiated with the highest dose. In contrast, EEG/Ag NP composites show a decreased contact angle compared to pristine EEG, and, similarly to GO, the highest value was measured for the composite irradiated with the highest dose. Although there are reports that the introduction of metallic nanoparticles can significantly affect the graphene’s wetting properties [44,45], the observed differences between GO and EEG composites might also be attributed to the film preparation method, which introduces a large number of wrinkles, pinholes, and micro-cracks to the film’s surface that might alter its structural properties.

**Figure 8:**
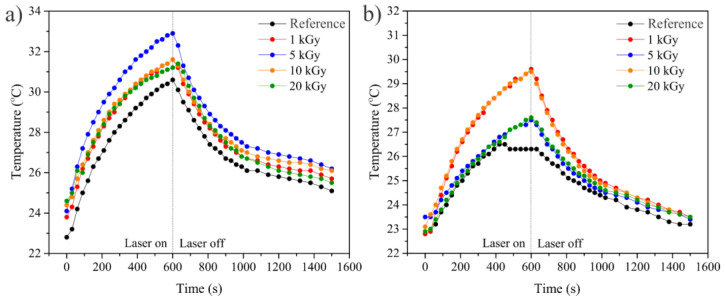
The temperature changes induced by a 532 nm laser irradiation for (**a**) GO (reference) and GO/Ag NPs prepared at different irradiation doses and (**b**) EEG (reference) and EEG/Ag NPs prepared at different irradiation doses.

To measure the light-to-heat conversion efficiency, GO/Ag NPs and EEG/Ag NPs samples were optically stimulated by a 532 nm continuous wave laser irradiation simultaneously monitoring the temperature elevation. After reaching the temperature plateau, the laser was switched off, letting the sample spontaneously cool down to ambient temperature and monitoring the temperature in the cooling phase. Based on these heating–cooling temperature profiles (Figure 8), we evaluated the efficiency of Ag NPs prepared on GO and EEG to induce the local temperature increase under photostimulation. Both GO and EEG without the presence of Ag NPs show a temperature increase upon laser illumination. Due to the presence of delocalized p-electrons in graphene’s sp^2^ lattice, graphene-based materials are capable of converting incident light into thermal energy and transferring it to the surrounding medium [46]. For GO/Ag NPs, the highest temperature increase (10.6 °C) was measured for the sample prepared at a 5 kGy dose, while for EEG/Ag NPs, the highest increase in temperature of 7.3 °C was measured for the samples prepared at 1 and 10 kGy irradiation doses. Considering the sample absorbance at the laser wavelength, the specific heat capacity of water (4.186 J/g°C) and graphene (0.72 J/g°C), the temperature maximum achieved for pure water (25.2 °C), and applied laser power, we calculated the photothermal efficiency for GO/Ag NP and EEG/Ag NP composites (Table 2). As expected, the highest calculated photothermal efficiency for graphene/Ag NP composites had a good correlation with the highest measured temperature increase upon laser illumination. 

**Figure 9:**
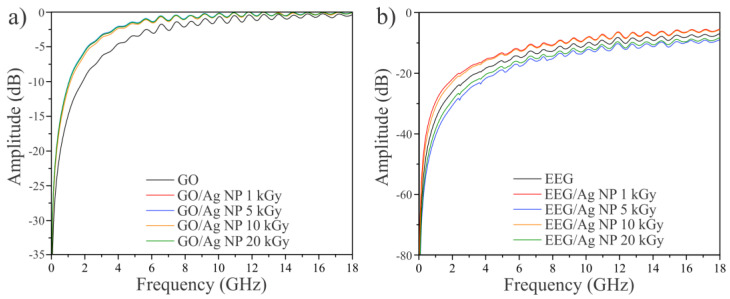
EMI shielding efficiency measurements. (**a**) S_21_ amplitude of GO and GO/Ag NPs composites, and (**b**) S_21_ amplitude of EEG and EEG/Ag NPs composites.

The EMI shielding performance of the pristine graphene-based materials and prepared composites was estimated by measuring the amplitude of the *S*-parameters (Figure 9). As can be seen, the transmission coefficient of the GO did not show any difference with the reference signal (paper), which indicates the poor EMI shielding efficiency of GO. Similarly to GO, GO/Ag NP composites showed poor EMI shielding efficiency. On the other hand, the EEG sample showed a difference between the reference and the loaded measurement. At 2 GHz, the difference in the amplitude of the transmission coefficient S_21_ was around 10 dB, which corresponds to 32% of the RF power transmitted through the sample. Contrary to GO/Ag NP composites, all samples prepared with EEG showed EMI shielding to some extent, and the highest values were measured for the composites prepared at 5 and 20 kGy doses.

## 4. Conclusions

We employed low-dose gamma irradiation (1–20 kGy) to prepare nanocomposites of silver nanoparticles anchored to two types of graphene-based materials: graphene oxide and electrochemically exfoliated graphene. The prepared Ag nanoparticles were spherical and homogeneously distributed onto the graphene sheets. Their predominant size distribution was 10–50 nm for graphene oxide and 10–100 nm for electrochemically exfoliated graphene. The photothermal efficiency measurement showed the highest photothermal efficiency of 29.5% for the graphene oxide/Ag NP samples prepared at 5 kGy. EEG/Ag NPs composites showed improved EMI shielding properties compared to their GO counterparts.

## Figures and Tables

**Table 1:** Elemental composition of GO, EEG, and their composites with Ag nanoparticles prepared at different irradiation doses.

Sample	Wt.%			Sample	Wt.%		
	C	O	Ag		C	O	Ag
GO	70.9	29.1		EEG	76.7	23.3	
GO/Ag NP 1 kGy	62.6	28.7	8.7	EEG/Ag NP 1 kGy	84.9	14.3	0.8
GO/Ag NP 5 kGy	63.0	29.0	8.0	EEG/Ag NP 5 kGy	82.5	13.0	4.5
GO/Ag NP 10 kGy	60.6	25.8	13.6	EEG/Ag NP 10 kGy	74.4	16.9	8.7
GO/Ag NP 20 kGy	63.3	21.9	14.8	EEG/Ag NP 20 kGy	78.0	15.2	6.8

**Table 2:** The calculated photothermal efficiency for GO/Ag NP and EEG/Ag NP composites.

Sample	Photothermal Efficiency (%)	Sample	Photothermal Efficiency (%)
GO	13.8	EEG	4.7
GO/Ag NP 1 kGy	23.1	EEG/Ag NP 1 kGy	20.8
GO/Ag NP 5 kGy	29.5	EEG/Ag NP 5 kGy	7.6
GO/Ag NP 10 kGy	15.9	EEG/Ag NP 10 kGy	15.2
GO/Ag NP 20 kGy	23.8	EEG/Ag NP 20 kGy	7.0

## Data Availability

Datasets analyzed in the current study are available in the Zenodo repository (https://doi.org/10.5281/zenodo.10053980 accessed on 25 April 2024).
